# PfMDR1 Transport Rates Assessed in Intact Isolated *Plasmodium falciparum* Digestive Vacuoles Reflect Functional Drug Resistance Relationship with *pfmdr1* Mutations

**DOI:** 10.3390/ph15020202

**Published:** 2022-02-07

**Authors:** Nina Simon, Cornelia Voigtländer, Barbara Kappes, Petra Rohrbach, Oliver Friedrich

**Affiliations:** 1 Institute of Medical Biotechnology, Friedrich-Alexander University Erlangen-Nürnberg, Paul-Gordan-Str. 3, 91052 Erlangen, Germany; nina.simon@fau.de (N.S.); cornelia.voigtlaender@fau.de (C.V.); barbara.kappes@fau.de (B.K.); 2 Institute of Parasitology, McGill University, Montreal, QC H3A 1B1, Canada

**Keywords:** drug resistance, *Plasmodium falciparum*, PfMDR1, P-glycoprotein 1, Fluo-4, transport protein

## Abstract

Drug resistance often emerges from mutations in solute transporters. Single amino acid exchanges may alter functionality of transporters with ‘de novo’ ability to transport drugs away from their site of action. The PfMDR1 transporter (or P-glycoprotein 1) is located in the membrane of the digestive vacuole (DV), functions as an ATP-dependent pump, and transports substrates into the DV. In this study, four strains of *Plasmodium falciparum*, carrying various *pfmdr1* gene mutations, were analysed for their transport characteristics of Fluo-4 in isolated DVs of parasites. To obtain quantitative estimates for PfMDR1 DV surface expression, PfMDR1 protein amounts on each strain’s DV membrane were evaluated by quantitative ELISA. Fluo-4, acting as a substrate for PfMDR1, was applied in DV uptake assays (‘reverse Ca^2+^ imaging’). Viable DVs were isolated from trophozoite stages with preserved PfMDR1 activity. This newly developed assay enabled us to measure the number of Fluo-4 molecules actively transported into isolated DVs per PfMDR1 molecule. The drug-resistant strain Dd2 presented the highest transport rates, followed by K1 and the drug-sensitive strain 3D7, compatible with their copy numbers. With this assay, an evaluation of the probability of resistance formation for newly developed drugs can be implemented in early stages of drug development.

## 1. Introduction

*Plasmodium falciparum* (*P. falciparum*) is most prevalent on the African continent and, as the cause of malaria, continues to be a major cause of death, in particular in children under the age of five [1]. Drug resistance has been documented in all approved anti-malarials to date. Treatment options have become difficult, and new drug development is essential and requires testing in appropriate preclinical assays. An example for potential new generation compounds would be the formation of drug-hybrids to prevent fast resistance development [2,3]. Most common assays currently employ in vitro parasite growth assays and focus on the global readout of parasitic proliferation in infected red blood cells (iRBC) by detection of DNA [4] or metabolic activity [5]. These assays are cumbersome and may not unravel specific targets within parasite organelles [6].

During its blood cycle, the malaria parasite infects human red blood cells (RBCs) and successfully evades the human immune system. For nutrient uptake within the confined RBC milieu, the pathogen builds up a transport system and digests the host’s haemoglobin in the digestive vacuole (DV), producing the bio-inert crystalline hemozoin. Since intermediates of the haemoglobin digestion pathway in the DV are toxic for the parasite [7], drugs are often directed against these metabolic steps. Spontaneous mutations in genes encoding for solute and drug transport proteins potentially expand their substrate repertoire and, consequently, promote new drug resistances. One crucial resistance against, for instance chloroquine, resulted in drug efflux activity developed by mutations in PfCRT (chloroquine resistance transporter). Along with this transporter, the DV membrane possesses an additional multi-domain protein associated with drug resistances, i.e., PfMDR1 (multi-drug resistance protein 1), also referred to as P-glycoprotein homologue 1 (Pgh1) [8].

A homologue of the parasitic PfMDR1 is a pleiotropic human ABC transporter that is associated with drug resistance in cancer cells [9,10] and named permeability glycoprotein (P-gp). P-gps transport a wide variety of substrates across extra- and intracellular membranes. Cancer cells express large amounts of P-gp, further amplifying effects in multidrug resistances [11].

**Figure 1:**
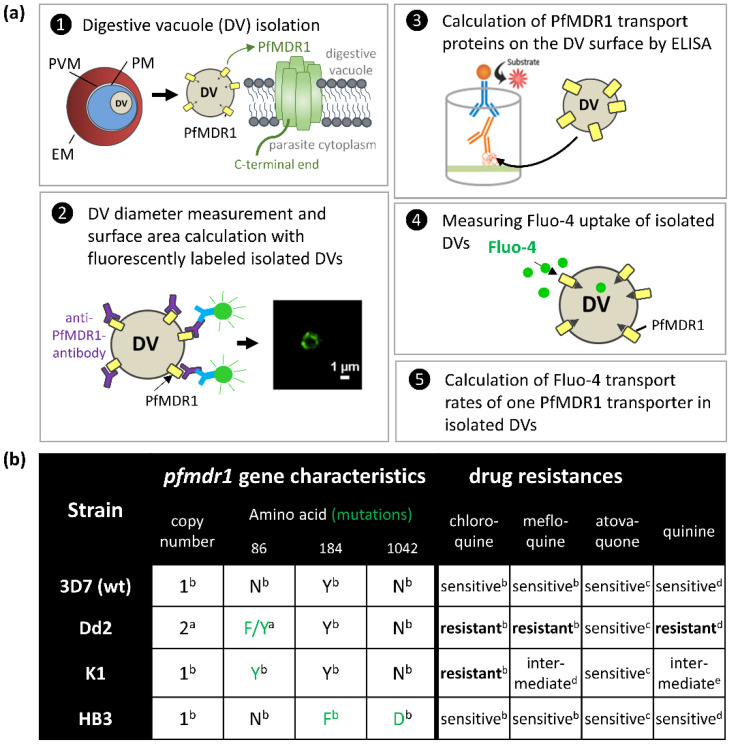
Bioprocess sequence and *P. falciparum* strain characteristics. (**a**) For the calculation of Fluo-4 transport rates of the multidrug resistance transporter PfMDR1, digestive vacuoles (DV) were isolated from four different *P. falciparum* strains (listed in (**b**)). The DV is surrounded by the erythrocyte membrane (EM), the parasitophorous vacuolar membrane (PVM), and the parasite membrane (PM) [14]. Antibodies used in this study were directed against a short peptide sequence at the C-terminal end of the PfMDR1 protein that is facing the parasite cytoplasm (1). The diameter of isolated DVs was microscopically measured with fluorescently labelled DVs (2). For the calculation of the number of PfMDR1 transporters on the surface of DVs from different parasite strains, recombinant proteins were produced. This amino acid sequence reflects the C-terminal end of the PfMDR1 transporter against which the anti-PfMDR1 antibody is directed. ELISA signals of lysed DVs were compared with a standard curve from recombinant PfMDR1 proteins to calculate the amount of PfMDR1 transporters on a defined DV surface area (3). Since the fluorescent dye Fluo-4 is a substrate of the PfMDR1 transporter, uptake assays with isolated DVs were performed (4) to calculate Fluo-4 transport rates of single PfMDR1 transport proteins (5). (**b**) The table depicts the main *pfmdr1* gene characteristics and strain drug resistances. Each strain carries different mutations in the *pfmdr1* gene; only Dd2 holds two *pfmdr1* gene copies, one carrying phenylalanine (F) at position 86, and the second gene tyrosine (Y) at the same position. Dd2 and K1 feature the most resistances, HB3 and 3D7 are sensitive to the antimalarials listed here ^a^ [13] ^b^ [15] ^c^ [16] ^d^ [17] ^e^ [18].

The DV is highly shielded in its multi-compartment environment. It is surrounded by the RBC membrane, the parasitophorous vacuolar membrane, and the parasite membrane (Figure 1a). Due to the localization of PfMDR1 and its relative inaccessibility, detailed characterizations regarding its transport rates in endogenous organelles are elusive. Nevertheless, studies exist on P-gp transport rates in cancer cells [12]. Additionally, artificial MDR1 Ligand Screening Kits are on the market (Biovision # K507), but are not adapted for the plasmodial transporter, PfMDR1.

In particular, *in situ* assessment of PfMDR1 transport rates is precariously hampered by the aforementioned conditions. In one of our previous studies, we developed a ‘reverse Ca^2+^ imaging’ approach using Fluo-4 as a readout transport substrate for PfMDR1 rather than monitoring Ca^2+^ ions within a DV environment that was previously shown to be relatively stable in free Ca^2+^ levels across *Plasmodium* strains [10,13]. This ‘in situ’ study enabled us to provide first estimates on overall global PfMDR1 transport rates in the intact iRBC-DV system of *P. falciparum* parasites. This system, however, may still be prone to underlying differences in buffer capacitance within the respective compartments to decisively influence observed Fluo-4 transport rates in the intact iRBC system over a putative isolated DV approach.

The aim of the present study was, therefore, to develop an assay to assess PfMDR1 transport rates using viable DVs isolated from trophozoite stages of the parasite. For this, we describe a multi-step bio-separation approach to obtain isolated DVs from *P. falciparum* parasites and validate their viability by showing intact ATP-dependence of PfMDR1 transport using isolated DVs. Two drug sensitive strains, 3D7 and HB3, and two drug resistant strains, K1 and Dd2, of *P. falciparum* were characterized. All strains carry different *pfmdr1* gene mutations at distinct localizations in the amino acid sequence. As a special feature, Dd2 possesses two copy numbers of mutated *pfmdr1* ([13]; Figure 1b) and reflects, together with K1, most resistances compared to the other strains, HB3 and 3D7, of this study (Figure 1b).

Purified DVs, thus, reflect a useful system to test novel drugs and study mechanisms of drug resistance conversion that is not yet available in this form. To obtain molecular transport rates per molecule of PfMDR1, we followed a defined process that involved the assessment of PfMDR1 DV expression densities across *P. falciparum* strains in a novel quantitative approach (Figure 1a). First, DVs were isolated from parasite trophozoite stages with preserved functionality of PfMDR1. The diameter and thus, the surface area of isolated DVs of each strain were obtained for the final calculation of PfMDR1 protein per µm^2^. For better detection of DV membranes, PfMDR1 transporters were labelled by indirect immune fluorescence. Following this, the amount of PfMDR1 proteins was evaluated by ELISA. The signals of recombinant PfMDR1 proteins functioned as a standard curve and were compared with signals of DV lysates, both labelled with an anti-PfMDR1 antibody. Fluo-4 uptake assays were then used to measure the number of Fluo-4 molecules taken up by isolated DVs via the ATP-dependent PfMDR1 transporter in a high-content optical assay (Figure 1a).

Combining the surface area, the number of PfMDR1 transporters per area, and the Fluo-4 molecules taken up by isolated DVs, it was possible to calculate transport rates of a single transporter for each *P. falciparum* strain.

## 2. Results and Discussion

### 2.1. Surface Area of Isolated Digestive Vacuoles

**Figure 2:**
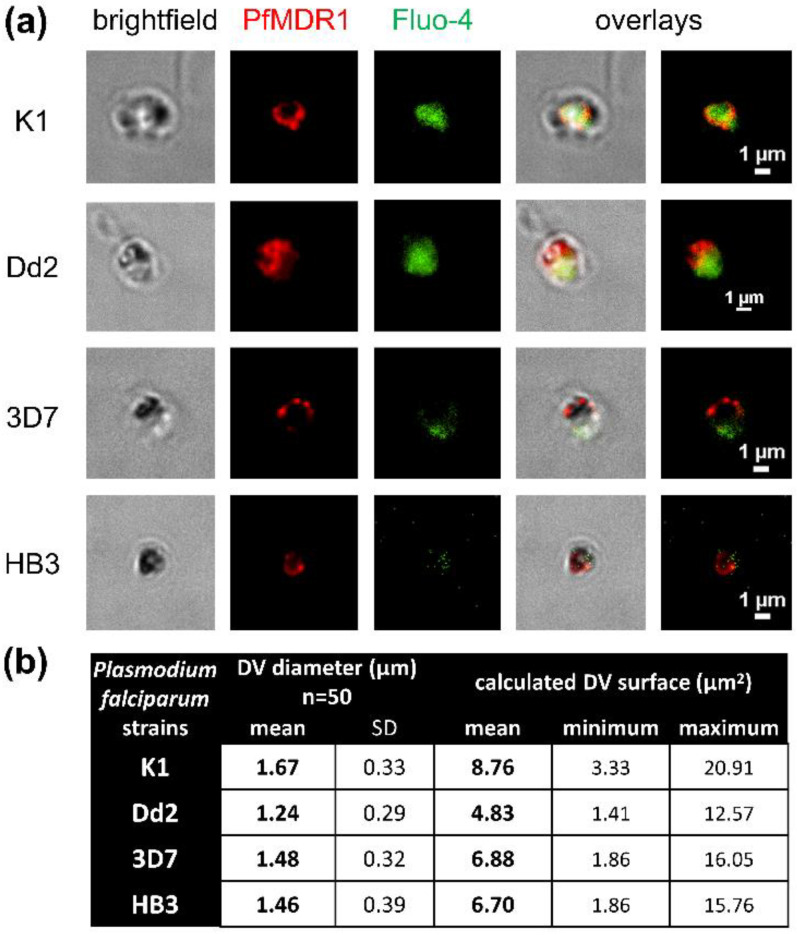
DV diameter measurement and surface area calculation. (**a**) DVs isolated from *P. falciparum* trophozoite stages were fluorescently labelled with anti-PfMDR1 antibodies to demonstrate PfMDR1 accessibility and label DV surface area to provide reference points for diameter measurements. (**b**) The surface area was calculated under the assumption of spherical DV shapes. n = number of measured DVs, SD = standard deviation.

The four strains of *P. falciparum*, K1, Dd2, 3D7, and HB3 were synchronized twice and harvested at the trophozoite stage to isolate digestive vacuoles. The organelles were used for Fluo-4 uptake assays and subsequently labelled with PfMDR1 antibodies for indirect immunofluorescence without membrane permeabilization (Figure 2a). This confirmed the accessibility of the DV membrane and PfMDR1 transporters. This confirmation was crucial to allow for future PfMDR1 transport studies. Additionally, fluorescent membrane labelling facilitated diameter measurements of the isolated organelles. With a 1.67 µm mean diameter, K1 has the largest DVs in this study (Figure 2b). With an assumed spherical shape, K1 DVs have a mean surface area of 8.76 µm^2^. Dd2 DVs are the smallest in this study with a mean surface area of 4.83 µm^2^, followed by HB3 and 3D7, which have similar surface areas. K1 and Dd2 also clearly showed Fluo-4 accumulated in their DVs, whereas 3D7 and HB3 show less fluorescence that was in accordance with predominantly passive diffusion (detailed data in Section 2.3, see also [13]).

### 2.2. Estimated PfMDR1 Transporter DV Surface Expression

**Figure 3:**
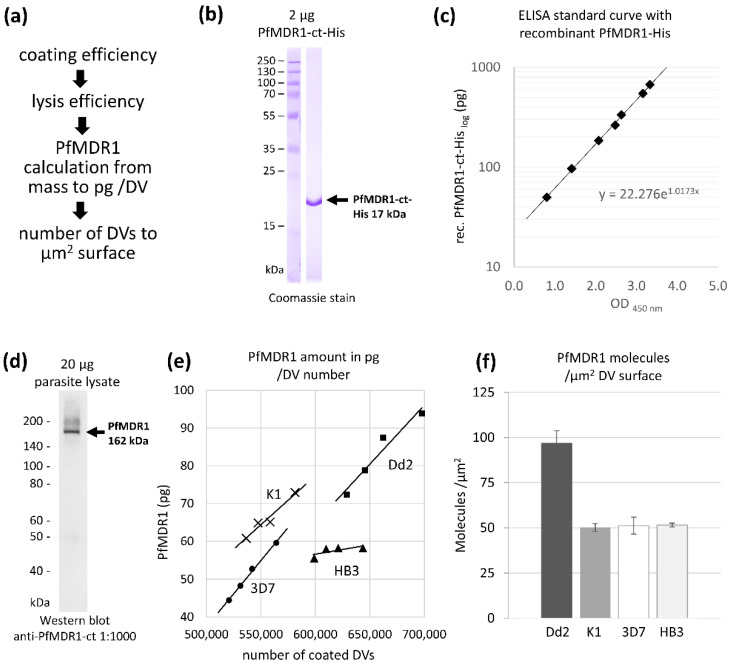
Calculation of PfMDR1 transport protein densities on the DV surface using ELISA. (**a**) For the calculation of the number of PfMDR1 proteins on a defined area, or on one DV, the following method was used: First the amount of total protein in one ELISA well was measured after coating the DV lysates. Then the lysis efficiency, which describes the amount of soluble PfMDR1 proteins present in the DV lysate, was defined by Western blot assay, comparing signals of total DVs with the supernatant of lysates. By the evaluation of DV lysates’ signals and standard curve signal, the total mass of PfMDR1 proteins was established and re-calculated to one DV. This was then downscaled to one µm^2^ of DV surface, with surface area data acquired from Figure 2. (**b**) Purified recombinant PfMDR1-ct-His protein on Coomassie gel. The target sequence is 17 kDa with a His-tag and was inserted for the generation of an ELISA standard curve. (**c**) The ELISA standard curve was generated with recombinant PfMDR1-ct-His proteins and detected with the anti-PfMDR1-ct-GST antibody (1:2000). (**d**) The specificity of the anti-PfMDR1-ct antibody was confirmed by Western blot assays on parasite lysate (dilution 1:1000, left) showing defined detection of the 162 kDa full-length protein without unspecific binding to other parasite proteins. (**e**) The amount of PfMDR1 proteins in the soluble fraction of DV lysate was evaluated by an indirect ELISA. The PfMDR1-containing DV proteins were coated onto an ELISA plate and detected with the anti-PfMDR1-ct antibody (1:2000) and goat-anti rabbit-HRP antibody (1:10,000). Calculation of PfMDR1 proteins in isolated DVs was compared with signals from wells coated with recombinant PfMDR1-His. Factoring the coating and lysis efficiency of the samples, Dd2 DVs contain the highest amount of PfMDR1 proteins in the samples compared to the other strains. (**f**) By incorporating the DV surface area data, Dd2 DVs express twice as many PfMDR1 molecules per µm^2^ surface than K1, 3D7, and HB3 DVs.

One important requirement for the calculation of Fluo-4 transport rates per molecule of transporter is the number of PfMDR1 proteins expressed on the surface of each DV. For this, an indirect ELISA, under denaturing conditions of the target protein PfMDR1, was developed. For ELISA calculations, a defined order of data integration was applied. Raw ELISA data were processed in the following order: First, the coating, then lysis efficiency (explanation see below) was considered, followed by PfMDR1 mass calculation per DV, with the final calculation of the number of transport protein on one µm^2^ DV surface (Figure 3a).

**Figure A1:**
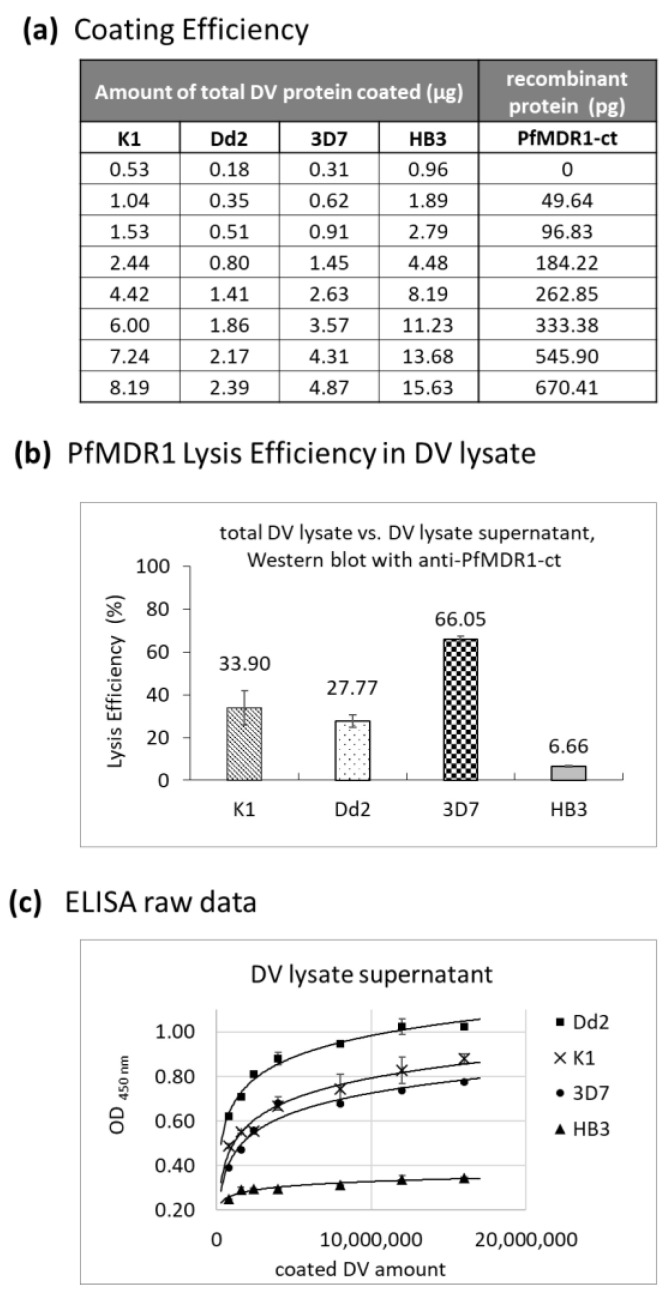
Coating and lysis efficiency, ELISA standard curve and raw data. (**a**) After lysis, soluble proteins from DV lysates were separated from insoluble components. The soluble part was coated on the ELISA plate and adhered total protein concentration was measured. The remaining coated protein mass was depicted in the table. (**b**) To determine the amount of soluble PfMDR1 proteins in the DV supernatant compared to whole DVs, the PfMDR1 protein was detected in a Western blot assay in both samples. About 34% of PfMDR1 proteins could be eluted from K1 DV lysate, 28% from Dd2, 66% from 3D7, and only approximately 7% out of HB3 DV lysate. (**c**) Raw ELISA data depict most PfMDR1 proteins out of Dd2 DV samples, followed by K1, 3D7 and HB3, without considering the coating or lysis efficiency. Absorption curves start to flatten at the fifth measurement point. Therefore, only the first four data point were used for quantification.

With an anti-PfMDR1 antibody [8] directed against a 17 kDa glutathione S-transferase (GST)-tagged peptide of the C-terminal (ct) end of PfMDR1 (Figure 1a), target proteins were readily detected. To generate a standard calibration curve, the PfMDR1-ct target amino acid sequence was recombinantly produced in *E. coli* with a His-tag and purified by Ni-NTA affinity purification (Figure 3b). As only a certain portion of the coated protein adheres to the ELISA wells surface, the coating efficiency of recombinant and endogenous protein had to be explicitly evaluated in advance (Figure A1a). The resulting calibration curve (Figure 3c) reflects defined signals of pure PfMDR1 proteins and was used to calculate PfMDR1 proteins in DV lysates of the four different strains of *P. falciparum*.

In addition to coating efficiency, the lysis efficiency of PfMDR1 proteins in DV lysates was evaluated. This defines the proportion of dissolved PfMDR1 proteins after DV lysis and can be detected in the supernatant of centrifuged DV lysates (Figure A1b). The best lysis efficiency was found in 3D7 DVs with 66% of PfMDR1 proteins found in solution, followed by 34% for K1 and 28% for Dd2. Exceptionally difficult to solubilize were PfMDR1 proteins from HB3 DVs. Here, only 6.7% of PfMDR1 proteins were found in the supernatant (Figure A1b).

Antibody specificity was initially confirmed on parasite lysate before its use in ELISAs. This protein mixture contains erythrocytic proteins, cell debris, cytosolic proteins, hemozoin, and DV proteins and was transferred to the Western blot membrane. The antibody did not detect proteins of this mixture in the range of 15 to 300 kDa; only the full-sized 163 kDa target protein PfMDR1 (Figure 3d) was labelled.

ELISA data, consisting of the standard curve and signals derived from DV lysates of all four *P. falciparum* strains, were compared and used to calculate the mass of PfMDR1 proteins in pg (Figure 3e). Together with Avogadro’s constant and the respective DV surface areas, it was possible to determine the number of PfMDR1 molecules per µm^2^ DV surface. Isolated K1, 3D7, and HB3 DVs contain approximately 50 PfMDR1 proteins per µm^2^ surface area, whereas Dd2 expressed twice as many transporters on the same area (Figure 3f). As is predominantly shown, duplicated genes also suggest protein expression doubling, as is the case here for Dd2.

### 2.3. Fluo-4 Uptake by PfMDR1 in Isolated DVs

Transport rate assessments by confocal microscopy using living cells involve elaborate sample preparations and time-consuming experimental procedures. The Fluo-4 uptake assay developed in [13] and optimized in this study allows the implementation of many experimental setups in parallel with sample material of consistent quality. A simple plate reader with a fluorescence measurement feature can measure billions of organelles in a few seconds in a high content screening environment. This is a major advantage compared to hours of microscopy sessions for the sequential measurement of single cells with a high-quality, expensive microscope, and potential loss of cell integrity of the samples during extended recording times, in particular when it comes to the photo-sensitivity of the DVs.

For the Fluo-4 transport rate evaluation of single functional PfMDR1 transport proteins, an assay with DVs isolated from trophozoite stages that imitates intracellular conditions was developed and optimized.

As Fluo-4 has been described as a substrate for PfMDR1 [10,13,19], it was used as a surrogate for heterocyclic aromatic drugs or other compounds that can be transported by PfMDR1. Because PfMDR1 is an ATP dependent transporter, we compared Fluo-4 uptake with and without the addition of ATP, to be able to (i) distinguish between active transport and diffusion, and (ii) document PfMDR1 functionality after DV isolation. Unfortunately, HB3 DVs could not be used for this assay because of incomplete lysis of surrounding cell membranes. Thus, HB3 DVs were excluded during assay performance. Different strains appear to have varying membrane stabilities, and HB3 parasites seem to have a much more stable parasitic and/or parasitophorous vacuole membrane than K1, Dd2, or 3D7 parasite strains. This was also evident in the lysis efficiency data for the ELISA. K1, Dd2 and 3D7 show approx. 27–66% lysis efficiency, whereas HB3 has only 6.7% when using the same lysis protocol. The difficulty in HB3 DV purification demonstrates that – in future assays – each strain might require an individually aligned protocol for DV isolation, followed by a quantitatively proven PfMDR1 accessibility and functionality.

**Figure 4:**
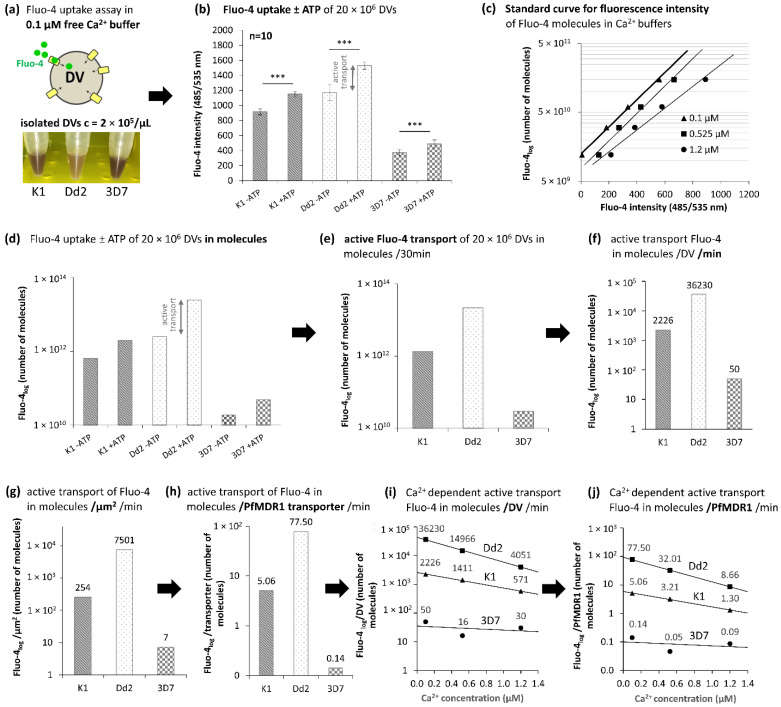
Fluo-4 uptake assays of isolated K1, Dd2 and 3D7 DVs (outlined for 0.1 µM free Ca^2+^ containing buffer). (**a**) DVs were isolated from *P. falciparum* trophozoites and (**b**) incubated with 10 µM Fluo-4 containing buffer with and without the addition of ATP. DV organelles were washed, and Fluo-4 that was transported into the DVs was measured with a Victor X4 plate reader (Perkin Elmer, Rodgau). The significance level (α) was set to 5% and p≤0.05 was considered significant. *p*-values were labelled as follows: ***-*p* ≤ 0.001. (**c**) For the calculation of the number of Fluo-4 molecules, a standard curve, with a defined number of Fluo-4 molecules, was generated using three buffers containing different free calcium (Ca^2+^) concentrations and one constant Fluo-4 concentration. (**d**) Fluorescence intensities of samples with ATP (+ATP) and without ATP (-ATP) were converted to Fluo-4 molecules. The difference between the -ATP and +ATP signals describes the active transport of Fluo-4 via the ATPase dependent PfMDR1 transporter [13]. (**e**) Fluo-4 uptake was measured with 20 × 10^6^ DVs after 30 min. (**f**) The number of transported molecules was re-calculated to one minute per DV assuming steady-state transport, (**g**) then per µm^2^ DV and afterwards (**h**) per PfMDR1 transporter, according to the ELISA data. (**i**) The assay was implemented in three buffers containing different Ca^2+^ concentrations (see Figure A2 for all three buffer diagrams and negative controls). Dd2 DVs produce the highest Fluo-4 uptake rates, followed by K1 and 3D7. Transport rates decrease with increasing Ca^2+^ concentrations. (**j**) One PfMDR1 transporter was able to pump approximately 77 Fluo-4 molecules per minute into an isolated Dd2 DV, followed by K1 with 5 molecules per minute, and 3D7 with 0.14 Fluo-4 molecules per minute.

**Figure A2:**
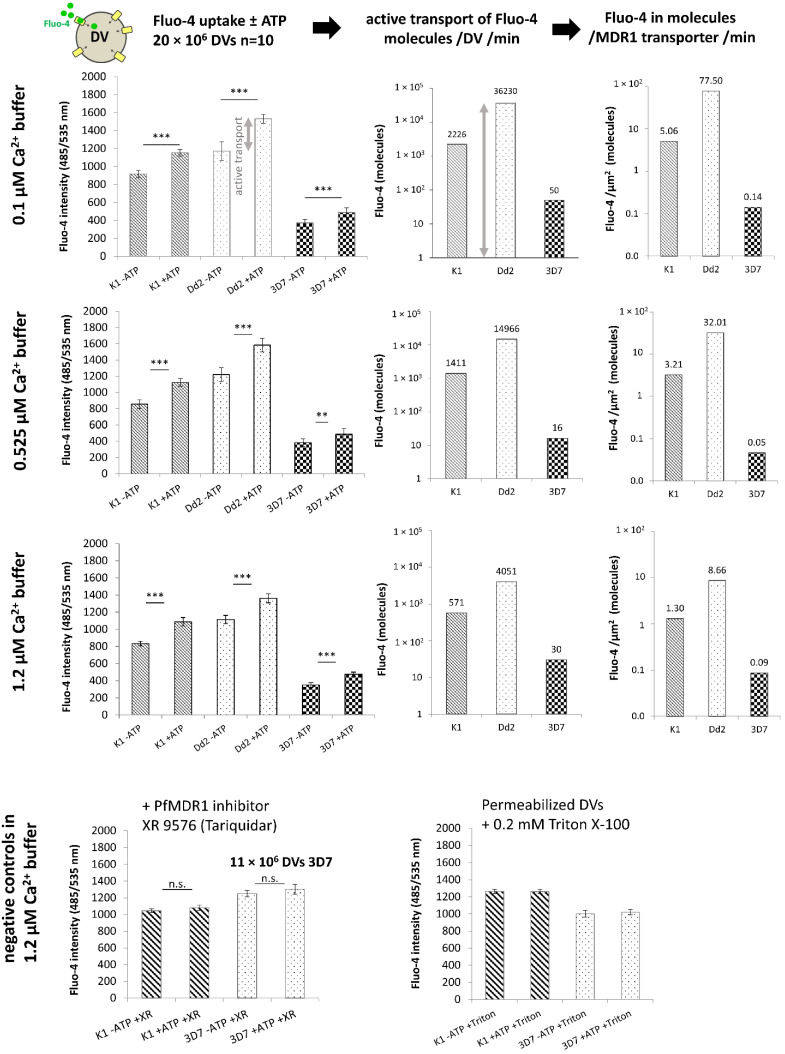
Fluo-4 DV uptake assays in three Ca^2+^ concentrations and negative controls. The detailed fluorescence datasets of all three Ca^2+^-containing buffers are depicted. Additional negative controls confirm specificity of Fluo-4 to the PfMDR1 transporter. Tariquidar is a specific PfMDR1 inhibitor and prevents the active uptake of Fluo-4 into K1 and 3D7 DVs. Triton X-100 perforated K1 and 3D7 DVs confirm no more active Fluo-4 transport into isolated DVs. The significance level (α) was set to 5% and *p* ≤ 0.05 was considered significant. *p*-values were labelled as follows: **-*p* ≤ 0.01; ***-*p* ≤ 0.001; n.s. = not significant.

As the fluorescence intensity of Fluo-4 increases both with a rise in total Fluo-4 and free Ca^2+^, we performed the assay in three buffers containing 0.1 µM, 0.525 µM, and 1.2 µM free Ca^2+^, respectively. For illustration purposes, only the 0.1 µM Ca^2+^-buffer data are depicted in Figure 4. The complete dataset with all three buffers can be found in Figure A2.

After assay implementation, fluorescence intensities of DVs were measured in white 96-well plates (see Methods). As the overall absorbance of hemozoin seems to differ between the strains (Figure 4a), potentially due to crystal sizes and associated Brownian movement [20], background signals may vary widely. In particular, the hemozoin of Dd2 DVs appears much brighter than DVs of other strains of *P. falciparum* and masks the reflecting white plate less. This can explain the apparent higher overall signal of Dd2 DVs (Figure 4b). It appears not to be the colour of hemozoin that differs; it might be the size of crystals, a different light refraction, or an increase in Brownian movement with different buffering capacities of the organelle compartment [20].

Of great importance is emphasizing the active transport of Fluo-4 by PfMDR1. This is shown by the difference in signal data found without ATP (-ATP) or with ATP (+ATP) present (Figure 4b). This also provides evidence for the viability of isolated DVs and their embedded PfMDR1 transporters, although a 100% preservation of PfMDR1 function cannot be concluded from the data.

For the correlation of Fluo-4 signals to the number of Fluo-4 molecules, three standard curves with different Ca^2+^ concentrations were generated (Figure 4c). The raw data of Figure 4c were converted to Fluo-4 molecules (Figure 4d). Mathematically, the difference between +ATP and −ATP fluorescence intensities describes the active transport portion of Fluo-4 translocation into the DV by PfMDR1. The number of Fluo-4 molecules being transported during 30 min into 20 × 10^6^ DVs of each strain (Figure 4e) can be converted to transport rates during one minute of Fluo-4 uptake (or pump activity) per DV (Figure 4f). It demonstrates that Dd2 transports the greatest number of Fluo-4 molecules compared to the other strains. With approx. 36,000 molecules per minute, the data are comparable to the ~50,000 Fluo-4 molecules measured using live cell microscopy of iRBCs [13].

Combining the surface data of the DVs (Figure 2b), the number of Fluo-4 molecules transported per µm^2^ DV surface (Figure 4g), and the number of PfMDR1 molecules per µm^2^ (Figure 3), Fluo-4 transport rates per PfMDR1 transporter were then calculated to be 5 molecules per minute for K1, 77 molecules per minute for Dd2, and 0.14 molecules per minute for 3D7. Of these three strains, only 3D7 has no amino acid substitution at position 86 (Figure 1b). 3D7 also showed the lowest PfMDR1 transport rates of Fluo-4 compared to Dd2 and K1. This could indicate that the amino acid at position 86Y leads to higher Fluo-4 transport rates of PfMDR1. Further, the significantly higher transport rates of Dd2 compared to K1 is likely due to the two copies of *pfmdr1* in Dd2 strains and possibly due to the amino acid exchange in one of the two gene copies at position 86 to phenylalanine (F) (Figure 1b).

As Fluo-4 is a Ca^2+^ indicator, its signal intensities increase with a rise in free Ca^2+^ present in the sample. This increase is recognized in the standard curves (Figure 4c). In the uptake assays, Fluo-4 transport rates decreased with increasing Ca^2+^ concentration (Figure 4i,j). However, with different Ca^2+^ concentrations, the number of Fluo-4 molecules being transported by one PfMDR1 should be consistent. Given the normal free Ca^2+^ concentration in malaria parasites is approx. 0.3 µM [19], the here implemented 0.525 µM and 1.2 µM might cause unknown effects on cell components. Potentially, the Fluo-4 transport efficiency, or binding capacity to PfMDR1 is influenced by increasing Ca^2+^ concentrations, either due to Ca^2+^ bound to Fluo-4, or Ca^2+^ bound to the DV membrane or PfMDR1 itself, or combinations thereof. Because each Fluo-4 molecule binds one Ca^2+^ ion [21], it might be possible that Fluo-4 without bound Ca^2+^ is also better transported by PfMDR1. This potential unbound Fluo-4 may subsequently bind to Ca^2+^ already present in the DV.

For confirmation of Fluo-4 transport specificity, the PfMDR1 inhibitor [10,22] Tariquidar (=XR9576) was incubated together with K1 and 3D7 DVs. No active Fluo-4 transport (DV fluorescence uptake) was detected in either strain (Figure A2). Furthermore, membrane-perforated DVs did not show any active Fluo-4 uptake (Figure A2), confirming that active transport relies on intact, viable DVs.

In summary, we provide the first characterization of molecular transport rates from PfMDR1 transporters embedded in their natural environment of the intact DV isolated from *P. falciparum* iRBCs for drug-sensitive and-resistant strains. This new assay will be of high value for future preclinical drug screening attempts involving novel malarial lead compounds.

## 3. Materials and Methods

### 3.1. Cultivation and Synchronization of Malaria Parasites

The four *P. falciparum* parasite strains, K1, Dd2, 3D7 and HB3, were cultivated in vitro in RPMI 1640 medium containing 2 mM L-glutamine, 25 mM 4-(2-hydroxyethyl)-1-piperazineethanesulfonic acid (HEPES) and 24 mM NaHCO_3_ supplemented with 0.1 mM hypoxanthine, 25 μg/mL gentamicin and 0.5% AlbuMAX^®^ I [23] containing 5% (*v*/*v*) A+ erythrocytes. Cultures were kept in an atmosphere of 5% O_2_, 5% CO_2_, and 90% N_2_ at 37 °C. Under these conditions, the Dd2 strain has a cycle length of 42 h (hours), 3D7 44 h, HB3 45 h, and K1 47 h to fulfil one asexual replication cycle. Dd2 and 3D7 strains were synchronized twice with 5% Sorbitol in a time interval of 11 h, whereas HB3 and K1 need an interval of 8 h to reach approximately same yields as Dd2 and 3D7 in the final purification step. The 48-h replications factor for Dd2 and 3D7 strains is about 8, whereas HB3 and K1 reproduce approximately 5 times in the same time frame. For DV isolation, trophozoite stages of Dd2 were harvested 53 h after the second synchronization and 3D7 55 h later. HB3 was harvested after 62 h, and K1 64 h after the second synchronization. The parasitaemia reached 5–6% when DV isolation was performed.

### 3.2. Isolation of Trophozoites and Digestive Vacuoles from Infected Red Blood Cells (iRBC)

*Trophozoite isolation*: A 1 mL sized iRBC culture pellet (equivalent to 5% RBC in 20 mL medium) was centrifuged in one 50 mL tube at 800× *g* for 3 min. The cell pellet was washed once in 20 mL phosphate-buffered saline (PBS) pH 7.2 and centrifuged again at 800× *g* for 3 min. Erythrocyte membranes were permeabilized by 10 min exposure to saponin (0.03% *w*/*v*) in 40 mL PBS. The tube was inverted and vortexed after 5 min incubation time for 10 sec. Isolated trophozoites were centrifuged with 2000× *g* at 4 °C for 5 min, and the supernatant (SN) was discarded. This step was repeated once, and cells were resuspended thoroughly in 10 mL PBS. Cells were counted in a Neubaur counting chamber. Counting cells at this point, instead of counting isolated DVs later, is more accurate because isolated DVs often stick together, which hinders precise counting. Remaining cells were stored on ice during counting, then isolated trophozoites were centrifuged at 2000× *g* for 5 min, and the SN was discarded.

*DV isolation* (partly inspired by [24]): For the lysis of parasite membranes, the parasite pellet was resuspended in ice-cold 0.05% Digitonin in 2 mL Uptake buffer (UB contains 120 mM KCl, 10 mM NaCl, 25 mM HEPES, 2 mM MgCl_2_, 5 mM Na_2_HPO_4_×2H_2_O, pH 7.2; mimics the parasite cytosol composition). The mixture was incubated for 4 min on ice. Then 1 mL UB containing 1 mg/mL bovine serum albumin (BSA) was added and centrifuged immediately at 4000× *g* at 4 °C for 5 min. The pellet was washed twice with 2 mL UB/1 mg/mL BSA and resuspended in 400 µL UB/1 mg/mL BSA/30% Glycerine containing 160 × 10^6^ DVs, respectively. Aliquots were frozen at −80 °C.

### 3.3. Immunofluorescence Labelling of Isolated DVs and Diameter Measurements

For diameter and surface measurements, remaining DVs from Fluo-4 uptake assays were reused. Consequently, these organelles already contain Fluo-4. To prove PfMDR1 accessibility and facilitate optical DV diameter measurements with Nikon NIS elements software, DVs were indirectly labelled with PfMDR1 antibodies without organelle perforation or fixation.

Approx. 200 × 10^6^ DVs were centrifuged for 2.5 min at 8000× *g* and resuspended in 1 mL UB. Organelles were blocked in 3% BSA/UB for 1 h at room temperature (RT). After centrifugation (settings as above), the DV pellet was resuspended and incubated with the primary antibody anti-PfMDR1-ct 1:200 in 500 µL 3% BSA/PBS at 4°C overnight. The next day, the DVs were centrifuged and washed three times in 1 mL UB. The organelles were then incubated with the secondary anti-rabbit antibody-Alexa 594 (dilution 1:1000, in 3% BSA/PBS) for 2 h at room temperature (RT). Finally, the labelled DVs were washed three times in PBS, resuspended in 200 µL UB, and DV diameters (d) were measured with 1000x magnification using a 100× oil immersion objective with an Eclipse T_i_ microscope (Nikon, Düsseldorf). The organelles were confined between a microscopic slide and a coverslip (1.5H) in UB buffer. For each parasite strain, 50 DVs were examined. The surface area (A) of spherical DVs was calculated with the formula A = π·d^2^.

### 3.4. Recombinant PfMDR1-c-Terminal-His Protein Production

To generate a standard curve in the ELISA assay with defined signals on known numbers of PfMDR1 (ID: PF3D7_0523000) molecules, a recombinant protein that covers the C-terminal (ct) directed target sequence of the anti-PfMDR1-ct-antibody was produced in *E. coli*. The antibody was generated in rabbits immunized with a 17 kDa recombinant PfMDR1-ct protein with a GST-tag [8]. To avoid cross reaction of our recombinant protein with antibodies directed against the GST-tag, we decided to produce the PfMDR1-ct protein with a His-tag.

*Generation of PfMDR1-ct-His in* E. coli: The following primers were implemented to multiply copies of the target gene by PCR: Fwd-*Bam*HI + *Nco*I: 5′-TAA TAA GGA TCC *ATG* GTA GTT ACT CAA GAA CCC ATG TTA TTT AAT ATG; and Rev-*Xho*I: 5′-TAA TAA CTC GAG TTT AGC TAA TTT TAC ATA TTT TTT ATA TAT TCC ATC TTG-3′. First, the PCR product was cloned into *pBluescript* II SK (+) plasmid and then into the final His-tag containing pET-21d(+) vector. The protein was produced in *E. coli* BL21 (DE3) bacteria. Protein expression was induced with 0.05 mM Isopropyl β-D-1-thiogalactopyranoside (IPTG) for 18 h at 18 °C.

*Protein purification:* As the major portion of the protein was insoluble, the following nickel-charged nitrilotriacetic acid (Ni-NTA) agarose purification was implemented under denaturing conditions. The lysis buffer with additives (LB+1) contains 50 mM Tris, 300 mM NaCl, 1 mM Benzamidine, 1 mM phenylmethylsulfonyl fluoride (PMSF), and 8 M urea at pH 8. An *E. coli* cell pellet of 100 mL bacteria culture was lysed with 5 mL LB+ and sonicated on ice water. After 10 min centrifugation at 18,000× *g* at 4 °C, the SN was mixed with 1 mL Ni-NTA agarose. Previously, 2 mL 50% agarose was washed once with 10 mL water and once with 10 mL WB (Wash Buffer with 50 mM Tris, 300 mM NaCl, 8 M urea, and 50 mM imidazol). For binding of proteins to the agarose column, the SN was restocked with 50 mM imidazol and loaded. The agarose-protein complex was washed three times with 8 mL WB. PfMDR1-ct-His-proteins were eluted seven times with 500 µL elution buffer (WB with 500 mM imidazol) each. Because the elutions showed highest purity (data not shown), all seven elutions were combined and stored in aliquots at −80 °C.

### 3.5. ELISA for the Detection of Recombinant and Endogenous PfMDR1

One 160 × 10^6^ DV aliquot was taken from −80 °C and thawed in a 37 °C water bath for 2 min. The DVs were then put on ice and centrifuged at 8000× *g* for 3 min at 4 °C. The supernatant was discarded, and DVs were resuspended in 400 µL LB+2 (8 M urea, 50 mM Tris, and 300 mM NaCl at pH 8). Then, the lysate was sonicated and centrifuged at 18,000× *g* for 10 min at 4 °C. The supernatant was implemented for lysis efficiency experiments together with total DV lysate in Western blot assays (Figure A1b) and for ELISA plate coating. All wells were pre-coated with 50 µL LB- (50 mM Tris, and 300 mM NaCl at pH 8), then endogenous DV and recombinant PfMDR1 proteins were diluted in different concentrations in a final volume of 50 µL LB+2 and added to all wells. This generates a final coating buffer containing 4 M urea/Tris/NaCl (also used as a Blank), which ensures a sufficient coating efficiency of recombinant and endogenous DV proteins after 1 h incubation at 37 °C (Figure A1a). Unbound protein was discarded, and all wells were washed once with 250 µL Tris buffered saline with 0.1% Tween (TBST). Remaining free protein binding sites were blocked with 110 µL 1% BSA in TBST for 1 h at RT. The plate was washed three times with 250 µL TBST, and 100 µL anti-PfMDR1-ct-antibody diluted 1:2000 in TBST was added for 1 h at RT to each well. The plate was washed again thrice with 250 µL TBST and incubated with 100 µL anti-rabbit antibody coupled to horseradish peroxidase (derived from goats) diluted 1:10,000 in TBST for 1 h at RT. The plate was then washed four times with 250 µL TBST and thoroughly tapped empty. 100 µL of TMB (Tetramethylbenzidine) substrate solution was added to each well and incubated for 30 min at RT. The enzymatic reaction was stopped with 100 µL stop solution (1N HCl). Absorptions were measured at 450 nm with a plate reader (Victor X4, Perkin Elmer, Rodgau, Germany).

### 3.6. Fluo-4 Uptake Assays with Isolated DVs

*Buffer preparation:* For Fluo-4 uptake assays, buffers containing three different free Ca^2+^ concentrations were prepared. For this purpose, the chelating agent ethylene glycol-bis(β-aminoethyl ether)-N,N,N′,N′-tetraacetic acid (EGTA) was implemented for free Ca^2+^ control. As the ATPase of PfMDR1 is at risk of being inhibited by EGTA concentrations higher than 0.07 mM [25], Ca^2+^ containing buffers were prepared with 0.01 mM EGTA, solely in plastic containers. Because the stability of free Ca^2+^ concentrations in this buffer is limited, strict incubation and preparation times must be followed. For this study, buffers were always prepared one day before the assay and used only on this day. Defined free Ca^2+^ concentrations were measured before assay implementation with a Fluo-4 calibration curve created with Calcium Calibration Buffer Kit #1 (#C3008MP, ThermoFisher, Dreieich, Germany). Two Fluo-4 incubation buffers (IB), one at pH 7.2 and one at pH 5.2 were required. They consist of 3 mM MgCl_2_, 100 mM KCl, 50 mM 3-(N-morpholino)propanesulfonic acid (MOPS), 50 mM Tris, without or with 2 mM ATP (+ATP, # R0441, ThermoFisher, [26]), without or with 10 µM Fluo-4 (+Fluo-4 Pentapotassium Salt, cell impermeant, # F14200, ThermoFisher, Dreieich, Germany), without or with 1 µM Thapsigargin (+TG, # T7459, ThermoFisher, Dreieich, Germany), and supplemented one day before with 0.01 mM EGTA with 1.2 µM (1.2Ca^2+^), 0.525 µM (0.525Ca^2+^), or 0.1 µM (0.1Ca^2+^). For 0.1 µM free Ca^2+^ buffer, no CaCl_2_ was added, this was the lowest Ca^2+^ concentration that could be reached with 0.01 mM EGTA. The assay was implemented in 0.5 mL tubes and measured in white polypropylene 96-well U-plates (Eppendorf, Hamburg, Germany).

*DV preparation:* In the same way as described for the ELISA, one DV aliquot from -80 °C was taken and thawed in a 37 °C water bath for 2 min. As many DVs as needed for the assay were combined and centrifuged at 8000× *g* for 3 min at 4 °C. DVs were treated again with 0.04% Digitonin in 2 mL uptake buffer (UB) containing 120 mM KCl, 10 mM NaCl, 25 mM HEPES, 2 mM MgCl_2_, 5 mM Na_2_HPO_4_x2H_2_O, pH 7.2 for 10 min at RT, then washed twice with 2 mL UB and kept on ice until used in uptake assays. Between thawing DVs and assay performance completion, not more than 2.5 h should elapse.

*Fluo-4 uptake assay*: One assay setup was composed of one Ca^2+^ concentration (three concentrations were implemented) and two parasite strains with 24 samples each. Here, the assay is described for 1.2 µM free Ca^2+^. For each strain, 10 samples with IB/1.2Ca^2+^/pH 7.2/+Fluo-4/-ATP, 10 samples with IB/1.2Ca^2+^/pH 7.2/+Fluo-4/+ATP and four samples only with IB/1.2Ca^2+^/pH 7.2 as a blank control were prepared. 20 × 10^6^ DVs for each sample were washed in 100 µL IB/0.1Ca^2+^/pH 7.2 and centrifuged at 8000× *g* for 3 min at 4°C. These centrifuge settings were used for all subsequent steps. The SN was discarded, and for Fluo-4 uptake, the pellet was resuspended in 75 µL IB/1.2Ca^2+^/pH 7.2/+Fluo-4/-ATP, or IB/1.2Ca^2+^/pH 7.2/+Fluo-4/+ATP, or solely in IB/1.2Ca^2+^/pH 7.2 and incubated for 30 min in a dark 37 °C water bath. All following steps were protected from light. Subsequently, 200 µL IB/1.2Ca^2+^/pH 7.2/+TG was added to the sample and then centrifuged. The supernatant was discarded and DVs were washed once in 100 µL IB/1.2Ca^2+^/pH 7.2/+TG and once in IB/1.2Ca^2+^/pH 5.2/+TG. Each sample was then taken up in 100 µL IB/1.2Ca^2+^/pH 5.2/+TG and transferred to an ELISA plate well. All samples were cooled down for 30 min to 4 °C, and Fluo-4 intensity was measured immediately in a plate reader with an excitation of 485 nm and emission of 535 nm with 0.1 s exposure time. For control setups, samples were incubated with 3 µM of the PfMDR1 specific inhibitor Tariquidar = XR-9576 and for perforation of DV membranes with 0.2 mM Triton X-100 [27].

For the entire study, one large batch of isolated DVs for each parasite strain was produced to ensure comparability between all data sets.

## 4. Conclusions

- Viable DVs from parasite trophozoite stages of RBCs infected with multi-drug resistant and sensitive strains can be obtained with an optimized bio-separation strategy described here.
- DVs isolated in this manner contain functional PfMDR1 transporters that are responsive to both ATP and drugs and can be used for ‘reverse Fluo-4 Ca^2+^ imaging’ to monitor PfMDR1 transport rates in the isolated compartment, free of cytoplasmic influences of the parasite.
- Amino acid exchanges due to mutations in the *pfmdr1* gene might lead to higher Fluo-4 transport rates of the PfMDR1 transport protein
- Isolated DVs can be subjected to a high-content assay that, alongside with morphometric analyses of DV surface area and quantitative ELISA for PfMDR1 binding studies, provide the unique opportunity to relate global transport rates from whole DVs to molecular PfMDR1 rates.
- The high content transport rate assay described here will be of high value for upcoming in vitro drug screening assays, as DVs can be stably stored following the bio-separation process and allow a constant sample quality.

## Data Availability

Data is contained within the article.

## References

[1] WHO (2020). World Malaria Report.

[2] Capci A., Lorion M.M., Wang H., Simon N., Leidenberger M., Borges Silva M.C., Moreira D.R., Zhu Y., Meng Y., Chen J.Y. (2019). Artemisinin-(Iso)quinoline Hybrids by C-H Activation and Click Chemistry: Combating Multidrug-Resistant Malaria. Angew. Chem. Int. Ed. Engl..

[3] Quadros H.C., Capci A., Herrmann L., D’Alessandro S., Fontinha D., Azevedo R., Villarreal W., Basilico N., Prudêncio M., Tsogoeva S.B. (2021). Studies of Potency and Efficacy of an Optimized Artemisinin-Quinoline Hybrid against Multiple Stages of the Plasmodium Life Cycle. Pharmaceuticals.

[4] Smilkstein M., Sriwilaijaroen N., Kelly J.X., Wilairat P., Riscoe M. (2004). Simple and inexpensive fluorescence-based technique for high-throughput antimalarial drug screening. Antimicrob. Agents Chemother..

[5] Makler M.T., Hinrichs D.J. (1993). Measurement of the lactate dehydrogenase activity of Plasmodium falciparum as an assessment of parasitemia. Am. J. Trop. Med. Hyg..

[6] Carolino K., Winzeler E.A. (2020). The antimalarial resistome—Finding new drug targets and their modes of action. Curr. Opin. Microbiol..

[7] de Villiers K.A., Egan T.J. (2021). Heme Detoxification in the Malaria Parasite: A Target for Antimalarial Drug Development. Acc. Chem. Res..

[8] Cowman A.F., Karcz S., Galatis D., Culvenor J.G. (1991). A P-glycoprotein homologue of Plasmodium falciparum is localized on the digestive vacuole. J. Cell Biol..

[9] Peel S.A. (2001). The ABC transporter genes of Plasmodium falciparum and drug resistance. Drug Resist. Updat..

[10] Rohrbach P., Sanchez C.P., Hayton K., Friedrich O., Patel J., Sidhu A.B., Ferdig M.T., Fidock D.A., Lanzer M. (2006). Genetic linkage of pfmdr1 with food vacuolar solute import in Plasmodium falciparum. EMBO J..

[11] Lehne G. (2000). P-glycoprotein as a drug target in the treatment of multidrug resistant cancer. Curr. Drug Targets.

[12] Sharom F.J. (2014). Complex Interplay between the P-Glycoprotein Multidrug Efflux Pump and the Membrane: Its Role in Modulating Protein Function. Front. Oncol..

[13] Friedrich O., Reiling S.J., Wunderlich J., Rohrbach P. (2014). Assessment of Plasmodium falciparum PfMDR1 transport rates using Fluo-4. J. Cell. Mol. Med..

[14] Matz J.M., Beck J.R., Blackman M.J. (2020). The parasitophorous vacuole of the blood-stage malaria parasite. Nat. Rev. Microbiol..

[15] Ding X.C., Ubben D., Wells T.N. (2012). A framework for assessing the risk of resistance for anti-malarials in development. Malar J..

[16] Chugh M., Scheurer C., Sax S., Bilsland E., van Schalkwyk D.A., Wicht K.J., Hofmann N., Sharma A., Bashyam S., Singh S. (2015). Identification and deconvolution of cross-resistance signals from antimalarial compounds using multidrug-resistant Plasmodium falciparum strains. Antimicrob. Agents Chemother..

[17] Nzila A., Mwai L. (2010). In vitro selection of Plasmodium falciparum drug-resistant parasite lines. J. Antimicrob. Chemother..

[18] Veiga M.I., Dhingra S.K., Henrich P.P., Straimer J., Gnadig N., Uhlemann A.C., Martin R.E., Lehane A.M., Fidock D.A. (2016). Globally prevalent PfMDR1 mutations modulate Plasmodium falciparum susceptibility to artemisinin-based combination therapies. Nat. Commun..

[19] Rohrbach P., Friedrich O., Hentschel J., Plattner H., Fink R.H., Lanzer M. (2005). Quantitative calcium measurements in subcellular compartments of Plasmodium falciparum-infected erythrocytes. J. Biol. Chem..

[20] Reiling S.J., Krohne G., Friedrich O., Geary T.G., Rohrbach P. (2018). Chloroquine exposure triggers distinct cellular responses in sensitive versus resistant Plasmodium falciparum parasites. Sci. Rep..

[21] Hamid E., Church E., Alford S. (2019). Quantitation and Simulation of Single Action Potential-Evoked Ca(2+) Signals in CA1 Pyramidal Neuron Presynaptic Terminals. eNeuro.

[22] Reiling S.J., Rohrbach P. (2015). Monitoring PfMDR1 transport in Plasmodium falciparum. Malar J..

[23] Trager W., Jensen J.B. (1976). Human malaria parasites in continuous culture. Science.

[24] Kehr S., Sturm N., Rahlfs S., Przyborski J.M., Becker K. (2010). Compartmentation of redox metabolism in malaria parasites. PLoS Pathog..

[25] Alonso G.L., González D.A., Takara D., Ostuni M.A., Sánchez G.A. (1998). Calcium additional to that bound to the transport sites is required for full activation of the sarcoplasmic reticulum Ca-ATPase from skeletal muscle. Biochim. Biophys. Acta (BBA)—Mol. Cell Res..

[26] Buxbaum E. (1999). Co-operating ATP sites in the multiple drug resistance transporter Mdr1. Eur. J. Biochem..

[27] Koley D., Bard A.J. (2010). Triton X-100 concentration effects on membrane permeability of a single HeLa cell by scanning electrochemical microscopy (SECM). Proc. Natl. Acad. Sci. USA.

